# Nosocomial SARS-COVID-19 Outbreak During the Third Wave of COVID-19 in an Oncology Facility at a Tertiary Care Hospital in Kashmir, India

**DOI:** 10.7759/cureus.43459

**Published:** 2023-08-14

**Authors:** Farhana Siraj, Mohmad Hussain Mir, Nazia Mehfooz, Karan Happa, Mushtaq Ahmad Sofi, Nisar Ahmad Syed, Faisal Guru, Saquib Z Banday, Nazir Ahmad Dar, Rafi A Jan

**Affiliations:** 1 Internal Medicine, Sher-i-Kashmir Institute of Medical Sciences, Srinagar, IND; 2 Medical Oncology, Sher-i-Kashmir Institute of Medical Sciences, Srinagar, IND; 3 Pulmonology, Sher-i-Kashmir Institute of Medical Sciences, Srinagar, IND; 4 Radiation Oncology, Sher-i-Kashmir Institute of Medical Sciences, Srinagar, IND; 5 Medical Oncology, State Cancer Institute, Sher-i-Kashmir Institute of Medical Sciences, Srinagar, IND

**Keywords:** sars-cov-2, immunocompromised, covid-19 illness, healthcare workers, nosocomial covid

## Abstract

Background: Coronavirus disease 2019 (COVID-19) emerged as a life-threatening respiratory condition, especially in immunocompromised patients, caused by severe acute respiratory syndrome coronavirus 2 (SARS-CoV-2). Initially detected in China in December 2019, the first case in India was diagnosed on January 30, 2020. Here we report a nosocomial COVID-19 outbreak among cancer patients and healthcare workers (HCWs) in a medical oncology unit of a tertiary care hospital from our region.

Materials and methods: This was a descriptive study of the nosocomial COVID-19 outbreak and was conducted in the month of January 2022 at the medical oncology unit of a tertiary care hospital in Srinagar, Jammu and Kashmir (J&K), India. The study included 25 COVID-19 cases, including patients and HC/non-HCWs (NHCWs). The confirmation of diagnosis was done through real-time polymerase chain reaction (RT-PCR) using nasopharyngeal/oropharyngeal swabs as the test sample.

Results: Twenty-five COVID-19 cases, including 14 admitted patients, nine HCWs, and two NHCWs were confirmed by COVID-19 RT-PCR in a span of 11 days. The first case was a positive HCW. The patients were admitted for management of various hematological as well as solid organ malignancies. Of the 14 patients, eight were in the pediatric age group with a mean age of 6.9 years, and six were adults with a mean age of 55.2 years. Thirteen patients were on different chemotherapy protocols, and one was undergoing an autologous stem cell transplant. Of the 14 patients, four were asymptomatic for COVID-19 symptoms, eight had mild disease, and two had severe disease with respiratory failure. Two patients with severe diseases needed COVID-19-designated high-dependency unit (HDU) admission. There was one COVID-19-related death. Among the healthcare workers, the mean age was 33.8 years, of which six were males and three were females. All the HCWs and NHCWs had mild disease, and all of them recovered completely.

Conclusion: Nosocomial COVID-19 illness is a new entity and is preventable. COVID-19 illness will remain in society after the pandemic is over, like the influenza B viral illness, and there can be seasonal flares in the future. Proper measures should be taken to prevent its clustering in hospital settings.

## Introduction

The novel coronavirus, severe acute respiratory syndrome coronavirus 2 (SARS-CoV-2), was discovered in Wuhan City, Hubei Province, China, in December 2019 [1,2]. This virus causes coronavirus disease (COVID-19), a respiratory illness associated with fever, cough, dyspnea, myalgia, and fatigue, with varying severity [3]. The first case of COVID-19 in India was reported on January 30, 2020 [4]. The first case in Jammu and Kashmir (J&K, a region in North India) was diagnosed on March 9, 2020 [5]. The Kashmir Valley in the region of J&K recorded its first case on March 17, 2020 [6]. COVID-19 was declared a pandemic by the World Health Organization (WHO) on March 11, 2020, and SARS-CoV-2 infections posed a serious threat to healthcare systems worldwide [7]. SARS-CoV-2 is the third member of the Coronaviridae family, along with severe acute respiratory syndrome coronavirus (SARS-CoV) and Middle East respiratory syndrome virus (MERS-CoV), to cause major epidemic outbreaks in the last 20 years [8,9]. Several studies demonstrate that SARS-CoV-2 is more contagious than SARS-CoV and MERS-CoV, and certain groups of patients are at high risk of COVID-19 infection due to a compromised immune system, high burden of comorbidities, and frequent hospitalization [10]. While the persistence of SARS CoV-2 has been described on inert surfaces, person-to-person transmission via droplets is believed to be the main mode of transmission followed by an incubation period of 5 days on average before onset of illness [11,12]. Several studies have recorded that approximately 50-60% of patients are asymptomatic [13]. The standard mode of diagnosis is testing nasopharyngeal or oropharyngeal swabs for SARS-CoV-2 nucleic acid using a real-time polymerase chain reaction (RT-PCR) assay [14,15].

Nosocomial COVID-19 includes infections that come directly from the environment through the healthcare worker's (HCW) hands or contaminated objects and as endogenous infection from patients, especially those who are immunocompromised and got infected by the virus at a hospital. The most important spreaders of nosocomial COVID-19 are the patients or the HCWs who are asymptomatic. Immune-compromised patients with mild symptoms spread the virus in the community through social interactions. Several publications have described nosocomial transmission during the COVID-19 pandemic [12,16]. Nosocomial outbreaks in different health centers have been described in several parts of the world [17-19]. In this study, we report an outbreak of COVID-19 among HCWs, non-healthcare workers (NHCWs), and inpatients at the medical oncology unit of our hospital. The aim was to determine the causes and characteristics of the outbreak and the measures implemented to prevent and control it. Here, we provide a detailed contact investigation including the clinical and laboratory findings of all individuals involved in the outbreak. Our observation emphasizes that nosocomial SARS-CoV-2 outbreaks require effective infection control strategies to prevent transmission within hospital settings.

## Materials and methods

The outbreak occurred in the month of January 2022 during the third wave of COVID-19 illness at a medical oncology ward in Sher-i-Kashmir Institute of Medical Sciences (SKIMS), a tertiary care hospital in Srinagar, J&K, India. The hospital has established separate care units and high-dependency areas for COVID-19 patients. A well-defined, protocol-based system was put in place for both the patients visiting the COVID-19 designated outpatient departments (OPDs) and for the patients who developed COVID-19 and presented in other non-COVID-19 wards in the hospital. The main aim of this system was to report this new and emerging entity and suggest measures to minimize the risks for both patients and HCWs. The ward where the outbreak occurred has 30 beds for inpatient admissions of different cancers, admitted for chemotherapy administrations and other complications of cancer and its treatment.

As per the hospital protocol, during this period only the visit of one relative was allowed per patient. The ward comprises 29 beds and a separate cabin for the transplant patient, one nursing station, two nurses' rooms, one nursing aid room, one storeroom, and one doctor’s consulting room. There are two points of entry into the ward. When the first case was confirmed, all 30 beds were occupied. The ward is well-ventilated with windows from all sides, but because of the sub-zero temperature in the valley during this period of the year (January 2022), most of the time, cross-ventilation is compromised in the ward because of the closed windows. The medical ward has 28 HCWs (doctors: four consultants, three medical oncology fellows, two internal medicine residents, four junior residents, ten nurses, and five nursing assistants) and two NHCWs (floor cleaners). The staff working in our ward followed COVID-19-appropriate behavior, (i.e., the use of N-95 masks, regular use of hand sanitizers, and notifying the patients as well as workers if they had any COVID-19-related symptoms). During the pandemic in general, and especially in the third wave of COVID-19, HCWs as well as the patients were advised to wear N-95 masks and clean their hands with alcohol-based hand sanitizers. The cases that developed the symptoms and were examined were inpatients, HCWs (doctors, nurses, nursing assistants), and non-HCWs (cleaning staff) of the unit. The confirmed cases are defined as inpatients, HCWs, or non-HCWs with a positive RT-PCR test for SARS-CoV-2 in the analysis of a nasopharyngeal/oropharyngeal swab. Samples were processed in the Department of Microbiology of our hospital.

Measurements

In this study, demographic characteristics were registered for each case, as well as previous comorbidities. In addition, we recorded their diagnosis, treatment with immune suppressants or not, Eastern Cooperative Oncology Group Performance Status (ECOG-PS) score, day of admission and the day on which they were tested as RT-PCR COVID-19 positive, the severity of their COVID-19 illness, oxygen requirements, and concomitant infections. Laboratory values of some basic investigations were also recorded, and X-ray findings and high-resolution computed tomography (HRCT) chest (if done) findings were also registered. The patients were divided into asymptomatic, mild, moderate, and severe COVID-19 illnesses as per their symptoms and in accordance with the All India Institute of Medical Sciences (AIIMS)/ Indian Council of Medical Research (ICMR) New Delhi COVID-19 National Task Force/Joint Monitoring Group of Director General of Health Services (DGHS) Ministry of Health and Family Welfare, Government of India CLINICAL GUIDANCE FOR MANAGEMENT OF ADULT COVID-19 PATIENTS revised on January 14, 2022. Patients with upper respiratory tract symptoms without hypoxia were treated as mild illness, patients with a respiratory rate of more than 24 per minute and oxygen saturation (SpO2) between 90-93% were categorized as moderate illness, and patients with a respiratory rate of more than 30 per minute and SpO2 less than 90% on room air were regarded as severe illness.

## Results

A total of 25 people were identified as COVID-19 RT-PCR positive in a span of 10 days from January 7 to January 17, 2022. These included 14 patients, nine HCWs, and two non-HCWs (Table 1).

**Table 1:** Global characteristics of COVID-19-positive patients HTN: hypertension; NHL: non-Hodgkin lymphoma; BHP: benign prostatic hyperplasia; CML: chronic myeloid leukemia; ECOG-PS: Eastern Cooperative Oncology Group performance status score; DOA: date of admission; VCR: vincristine; BFM-NHL: Berlin Frankfurt Munster non-Hodgkin lymphoma; DLBCL: diffuse large B-cell lymphoma; R-CHOP: Rituximab, cyclophosphamide, adriamycin, oncovin, prednisone; ALL: acute lymphoblastic leukemia; HR-1: high-risk phase one protocol; HDMTX: high-dose methotrexate; RT: radiotherapy; T2DM: diabetes mellitus type 2; PBF: peripheral blood film

Patient	Age	Sex	Comorbidity	Diagnosis	Myelosuppression/immunosuppression	Status of the disease	Concomitant active infection	ECOG-PS	DOA	RT-PCR+	N=day from admission on which patient came with COVID-19 +ve	COVID-19 severity	Death
1	3 months	Female	No	Wilms tumor	+/ Actinomycin and VCR	Not assessed	No	1	7/1/2022	13/1/2022	7	Mild	No
2	3 years	Male	No	Burkitts lymphoma	+/ CC Phase BFM-NHL-90 protocol	Relapse	No	1	11/1/22	17/1/22	7	Mild	No
3	55 years	Female	HTN, hypothyroidism, depression	Left breast DLBCL	+ / R-CHOP	Not assessed	No	2	7/1/2022	14/1/22	8	Mild	No
4	15 years	Male	No	Pre B-Cell ALL	+ /HR-1 BFM-95 ALL protocol	Relapse	No	1	5/1/2022	12/1/2022	8	Mild	No
5	55 years	Female	Hypothyroidism	Adenocarcinoma colon	-	Not applicable	No	2	9/1/22	14/1/22	6	Asymptomatic	No
6	75 years	Male	BHP	NHL	+/ Cyclophosphamide and prednisolone	Not assessed	No	3	10/1/2022	17/1/22	8	Severe	Yes
7	4 years	Female	No	Burkitts lymphoma	+/ Prephase V of BFM-90 NHL protocol	Not assessed	No	1	12/1/22	16/1/22	5	Mild	No
8	7 years	Male	No	T Cell ALL	+/ HD MTX	Responded	No	1	11/1/2022	15/1/2022	5	Mild	No
9	31 years	Male	No	CML with lymphoid blast crisis	+/ Dasatinib, steroids, VCR, danurubicin	Not assessed	No	1	1/1/2022	13/1/22	13	Asymptomatic	No
10	8 years	Male	No	ALL	+/BFM-95 ALL protocol	Not assessed	No	1	11/1/2022	16/1/22	6	Asymptomatic	No
11	40 years	male	No	Multiple myeloma	+/ Carfilzomib dexa and local RT	Not assessed	Yes	3	31/12/21	12/1/2022	13	Severe	No
12	5 years	Male	No	Neuroblastoma	+/ Post-stem cell transplant	Not assessed	Yes	2	13/12/21	14/1/22	32	Mild	No
13	5 years	Female	No	B-cell ALL	BFM-95 ALL protocol	D8 PBF negative	No	1	22/12/2021	15/01/22	25	Asymptomatic	No
14	75 years	Male	HTN, T2DM	Adenocarcinoma lung and CML (double malignancy)	Dasatinib	Not assessed	No	2	12/1/2022	17/1/2022	6	Mild	No

The first identified case was an HCW who tested positive on January 7, 2022. The number of patients who tested positive by RT-PCR was 14 (nine males and five females). During this period, 30 patients were admitted to the ward, out of whom 14 were COVID-19 positive. There are separate pediatric cubicles within the same ward, and among the 14 positive cases, there were eight in the pediatric age group and six in the adult age group. The average age in the pediatric group was 6.9 years, and the average age in the adult group was 55.2 years. Out of the 14 cases, five had hematological malignancies, which included B-cell acute lymphoblastic leukemia, T cell ALL, and chronic myeloid leukemia; eight had solid organ malignancies, which included Wilms tumor, colon cancer, and Burkitt’s lymphoma; and one patient had a double malignancy (i.e., lung adenocarcinoma with chronic myeloid leukemia). Out of eight patients in the pediatric age group, one had neuroblastoma and was admitted for an autologous stem cell transplant. The rest of the seven patients were admitted for chemotherapy administration. All six adult patients were admitted for chemotherapy. Out of 14 patients, 10 had ECOG PS-1, and two each had ECOG-PS 2 and ECOG-PS 3. Out of the COVID-19-positive cases, two had disease relapses. Among all the COVID-19-positive patients, five had leucopenia, two had neutropenia 1000/mm^3^, and four patients had a platelet count of less than 50,000/mm^3^ (Table 2).

**Table 2:** Investigation profile of the COVID-19-positive patients Hb: hemoglobin; TLC: total leukocytes; ANC: absolute neutrophil count; Bil: bilirubin; ALT: alanine transaminase; CXR: chest X-ray; HRCT: high-resolution computed tomography; B/l: bilateral; g/dl: gram per deciliter; mcl: per microliter; u/l: units per liter; mg/dl: milligram per deciliter

Patients	Hb(g/dl)	TLC/mcl	ANC/ml	Platelet/mcl	Creatinine (mg/dl)	Bil (mg/dl)	ALT (U/L)	CXR	HRCT chest
1	9.7	11.2	2912	507	0.2	0.19	24	Normal	Not done
2	10.5	10.2	6200	385	0.28	0.27	11	Normal	Not done
3	14.1	8.9	4500	145	0.44	0.7	18	Normal	B/l lower zone reticulations
4	9.8	9.1	9000	143	0.45	0.6	48	Normal	Not done
5	11.3	3.1	900	132	0.66	0.72	28	Normal	Not done
6	9.2	5.7	2850	41	0.86	0.5	30	B/l infiltrates	Not done
7	12.5	11.6	5500	309	0.4	0.22	20	Normal	Not done
8	12.8	3.7	1750	191	0.2	0.54	20	Normal	Not done
										9	10.1	2.9	1600	14	0.8	0.63	31	Normal	Not done
10	8.5	2.18	305	21	0.38	0.46	77	Normal	Not done
11	6.8	2.5	2000	14	1.94	1.31	94	B/l effusion and b/l lower zone infiltrates	B/l effusion, b/l lower zone consolidation
12	10.6	2.18	1300	19	0.2	0.68	24	Normal	Not done
13	8.7	1.8	600	75	0.45	0.62	27	Normal	Not done
14	12.1	10.4	8200	340	0.63	1.14	39	Lung lesion	Not done

With respect to the severity of the COVID-19 illness, out of 14 patients, four were asymptomatic for COVID-19 symptoms, eight had mild disease, and two had severe disease with respiratory failure. Two patients with severe diseases required high-dependency unit (HDU) admission. Among the two patients with severe COVID-19 illness, one had pulmonary nodular lymphoid hyperplasia (NHL) with bulky disease, and the other had multiple myeloma, severe anemia, and hypercalcemia. One patient had bilateral lower zone consolidation and pleural effusion on high-resolution computed tomography of the chest. There was one COVID-19-related death in the NHL case. Among the total 28 HCWs in the ward, nine HCWs tested COVID-19 RT-PCR positive; the mean age was 33.8 years, out of which six were males and three were females. All the HCWs and non-HCWs had mild disease, and all of them recovered completely (Table 3).

**Table 3:** Global characteristics of COVID-19-positive HCWs/non-HCWs PIVD: prolapsed intervertebral disc; T2DM: type 2 diabetes mellitus; RT-PCR: real-time polymerase chain reaction

Workers	Age	Sex	Comorbidities	RT-PCR COVID-19 positive	COVID-19 severity	Death
1	50	M	No	7/1/2022	Mild	No
2	30	F	No	15/1/22	Mild	No
3	27	M	No	14/1/22	Mild	No
4	26	M	No	14/1/22	Mild	No
5	26	M	PIVD	14/1/22	Mild	No
6	30	F	No	13/1/22	Mild	No
7	30	M	No	8/1/2022	Mild	No
8	50	M	T2DM	11/1/2022	Mild	No
9	55	M	No	12/1/2022	Mild	No
10	35	F	No	12/1/2022	Mild	No
11	30	M	No	13/1/11	Mild	No

## Discussion

In India, the COVID-19 situation was much worse during the second wave of illness. By the end of March 2022, India accounted for the second-highest number of confirmed cases after the USA. Since the first case of COVID-19 was reported in Wuhan City, the virus has evolved genetically over the last two years. In the present scenario, during the third wave of SARS-CoV-2, the variant of concern was B.1.1.529, designated as the Omicron variant by the WHO [20]. According to studies, the Omicron variant showed a 13-fold increase in viral infectivity, and it was 2.8 times more infectious than the Delta variant, which was the dominant strain during the second wave of COVID-19 [21]. The nosocomial COVID-19 outbreak is a well-defined entity and has been reported in various healthcare facilities globally during the pandemic [12,16-19]. Several studies provide evidence of the susceptibility of HCWs and NHCWs to be infected, especially after being exposed to patients initially not suspected of COVID-19, who are likely to transmit the virus at the presymptomatic or asymptomatic stages [22-27]. It has been estimated that >3,300 HCWs in China have been infected with SARS-CoV-2 during the outbreak, and in Italy, an estimated 20% of those infected were HCWs [28]. However, a detailed epidemiological characterization of transmission chains in the hospital setting remains scarce. Consequently, there is concern that infection control measures are not adequate to prevent SARS-CoV-2 transmissions between individuals in healthcare settings. As of January 25, 2022, the total number of cases in J&K was 409,166, including 47,376 active cases, 357,163 recoveries, and 4,627 deaths, and as of 11 February, the total number of cases was 449,873, including 7,424 active cases, 437,708 recoveries, and 4,741 deaths [29]. Our hospital is a multispecialty referral hospital with deemed university status, having more than 1,200 beds except for the oncology services, the rest of the hospital was designated as a COVID-19 hospital during the pandemic, and it caters to the population of eight million inhabitants in the region, besides other hospitals. Our unit, with a 30-inpatient bed capacity, is always fully occupied and will have immunocompromised patients the routine preventive measures may not be enough, and since immunocompromised patients are vulnerable to infections, special care and precautions must be put in place. Moreover, the source of spread in most of the cases remains unknown. With a number of social and cultural issues existing in our society, restricting the number of attendants per patient is a difficult task at times. Perhaps a way of screening the patients, their attendants, and HCWs and NHCWs prior to entering the units with vulnerable patients may help at times during the outbreaks or flares of the epidemic or pandemic illnesses. Infection control and disinfection strategies based on monitoring after the start of symptoms seemed insufficient to prevent transmission. In this outbreak, the scale of the problem included 32.14% of the HCWs working in the ward. All the HCWs had mild COVID-19 illness and fully recovered completely. Out of the 14 patients, only 14.3 % developed severe COVID-19 illness and needed admission to the COVID-19 high-dependency unit. Only one patient out of 14 cases was using bronchodilators from an aerosol-producing device. Even though the mortality rate in this outbreak was very low (one death only), it led to a delay in their existing chemotherapy regimens, which could have an effect on the prognosis of their basic disease. A similar outbreak of nosocomial COVID-19 occurred in a Spanish hospital in March-April 2020 in the medical ward for onco-hematological patients. Twenty-two COVID-19 cases (12 workers and 10 inpatients) were laboratory-confirmed. The initial cases were a healthcare provider and a visitor who tested positive. The median age of the patients was 73 years [13]. A similar COVID-19 outbreak happened in the pediatric dialysis unit of the University Hospital of Münster (Münster, Germany). A total of 48 COVID‑19 cases, including 28 healthcare professionals (HCPs), 13 patients, and seven accompanying persons (ACPs), were associated with a nosocomial infection. Among these cases, four COVID‑19 cases had 15-minute face-to-face contact with HCWs without wearing personal protective equipment (PPE), and seven other HCWs were infected while treating patients with COVID‑19 at a distance of less than 2 m without PPE [30]. Ventilation also has a role to play in the spread of COVID-19 in a hospital setting. A high concentration of the virus is found in poorly ventilated areas and sections of the hospital devoid of negative pressure. Another important aspect with regard to the spread of nosocomial COVID-19 is the use of aerosol-generating devices. The particles that are produced are less than 5 micrometers, and surgical masks cannot block such particles effectively. Therefore, in the pandemic setting, the use of such devices, especially in the hemato-oncological wards must be avoided. The major reason for the occurrence of nosocomial COVID-19 is the huge rush of attendants of the patients and HCWs who interact with the environment inside and outside the hospital. Another factor that contributes to these types of outbreaks is the fact that the patients needed to be shifted to different areas for various investigations, such as X-ray, computed tomography (CT) scans, radiotherapy sessions, etc., so the patient has significant contact with the external environment while shifting. The study analyzed the factors responsible for the spread of COVID-19 infection in non-COVID-19 areas in a hospital and the measures that can be instituted to prevent it. The asymptomatic COVID-19 cases neither seek care nor isolate themselves since they do not get tested in the first place, thus leading to a chain of spread that plays a pivotal role in these types of outbreaks. To prevent such outbreaks in the future, we suggest various measures to contain the spread and severity of nosocomial outbreaks (Table 4). 

**Table 4:** Measures to prevent nosocomial outbreaks of COVID-19 illness

Headings	Description							
Screening and epidemiological surveillance	The need for robust and active epidemiological surveillance, which, in addition to screening the patients with symptoms, should also screen the asymptomatic people who are at risk of having COVID-19 illness.							
	Regular screening of the attendants of the patients for the symptoms of COVID-19 illness and doing their routine screening for COVID-19 by RT-PCR.							
	Contact tracing of the COVID-19-positive patient or HCW is to be done by the community medicine department of the hospital in an active fashion so as to prevent further spread.							
	Doing a screening for COVID-19 by RT-PCR prior to admission for all the patients and repeating if any COVID-19 symptom develops. If a patient comes positive, all his/her contacts and the patient on adjoining beds, all HCWs who have been involved in the management should undergo testing and isolation as advised and modified from time to time.							
	Screening by simple questionnaires and if needed testing should be done at the time of admission in the wards.							
Prevention and Education	Restricting the number of attendants per patient would lead to a decrease in the exposure of the patient to indirect environmental exposure.							
	Regular use of N-95 masks or other recommended masks by the HCWs and the patients and attendants, use of alcohol-based hand sanitizers, and trying to maintain an appropriate distance from colleagues during ward rounds.							
	Refrain from using aerosol-generating devices like nebulizers or high-flow oxygen.							
	Improving the quality of ventilation (opening windows) in the wards and converting the hemato-oncological wards into negative pressure units in a similar manner as intensive care units.							
	For educational and awareness purposes, posters can be placed at different locations in the hospital emphasizing COVID-19-appropriate behavior.							
	Admission policy should be regulated during outbreaks and peak waves of illness.							
	The rooms should be more ventilated, no aerosols are generated, and only one attendant per patient wearing a mask should be allowed.							
Isolation and care	The designated areas for patients and professionals should be extensively cleaned due to the possibility of viral particles left on the surfaces returning to the air or to favor the spread via contact.							
	If any HCWs/non-HCWs develop any COVID-19 symptoms, he/she should be advised to isolate as recommended and do testing as per the latest ICMR guidelines.							
	Positive cases should be isolated and shifted to the designated COVID-19 HDU if severe illness is present.							

Limitations

It is difficult to know the source of infection in every case. Viral genome sequencing could have helped, but it was not available in our hospital at that time. Despite the aggressive testing for SARS-CoV-2, there may be undetected additional nosocomial cases due to false negative RTPCR test results or asymptomatic cases/HCWs were never tested. There is a lack of understanding about the risk of infection among HCWs and varied adherence to basic infection control practices, and it was not clear which practice was the most effective in preventing the transmission of SARS-CoV-2 in hospital settings.

## Conclusions

The COVID-19 pandemic has faded, but the illness is going to stay in society after the pandemic is over like the influenza B viral illness, and there will be seasonal flares. Nosocomial COVID-19 illness is a new entity and is preventable. Proper clinical evaluation of the patients for COVID-19-related symptoms and testing by RT-PCR, maintenance of COVID-19-appropriate behavior among the patients, attendants, and HCWs, and avoiding usage of aerosol-generating devices in the hospital setting, especially in sections where immune-compromised patients are admitted. These are a few measures that can be taken to prevent such outbreaks in hospital settings, especially in the areas where immunocompromised patients are admitted. As the pandemic has waned, there are warnings that similar or more severe pandemics will happen in the future, and it will be important to establish effective infection control strategies to protect frontline HCWs and patients from nosocomial infections.
